# *cheA*, *cheB*, *cheR*, *cheV*, and *cheY* Are Involved in Regulating the Adhesion of *Vibrio harveyi*

**DOI:** 10.3389/fcimb.2020.591751

**Published:** 2021-02-03

**Authors:** Xiaojin Xu, Huiyao Li, Xin Qi, Yunong Chen, Yingxue Qin, Jiang Zheng, Xinglong Jiang

**Affiliations:** ^1^ Fisheries College, Jimei University, Xiamen, China; ^2^ Engineering Research Centre of Eel Modern Industrial Technology, Ministry of Education, Xiamen, China; ^3^ Jimei University, Xiamen, China; ^4^ State Key Laboratory of Large Yellow Croaker Breeding, Ningde Fufa Fisheries Company Limited, Ningde, China; ^5^ Fujian Province Key Laboratory of Special Aquatic Formula Feed, Fujian Tianma Science and Technology Group Co., Ltd., Fuzhou, China

**Keywords:** adhesion, chemotactic gene, environmental factors, RNAi, *Vibrio harveyi*

## Abstract

Diseases caused by *Vibrio harveyi* lead to severe economic losses in the aquaculture industry. Adhesion is an important disease-causing factor observed in bacteria with chemotactic activity. In our study, we measured the adhesion of *V. harveyi* by subjecting the bacteria to stress using Cu^2+^, Pb^2+^, Hg^2+^, and Zn^2+^. The genes responsible for chemotaxis (*cheA*, *cheB*, *cheR*, *cheV*, and *cheY*), which are also crucial for adhesion, were identified and silenced *via* RNAi. We observed that a decrease in chemotactic gene expression reduced the ability of the organism to demonstrate adhesion, motility, chemotaxis, and biofilm formation. Upon comparing the *cheA*-RNAi bacteria to the wild-type strain, we observed that the transcriptome of *V. harveyi* was significantly altered. Additionally, the expression of key genes and the adhesion ability were affected by the pH (pH of 5, 6, 7, 8, and 9), salinity (NaCl at concentrations of 0.8, 1.5, 2.5, 3.5, or 4.5%), and temperature (4, 15, 28, 37, and 44°C) of the medium. Based on these results, the following conclusions were made: (1) The chemotactic genes *cheA*, *cheB*, *cheR*, *cheV*, and *cheY* may regulate the adhesion ability of *V. harveyi* by affecting bacterial motility, and participate in the regulation of adhesion at different temperatures, salinities, and pH values; (2) stable silencing of *cheA* could alter the transcriptional landscape of *V. harveyi* and regulate the expression of genes associated with its adhesion mechanisms.

## Introduction

*Vibrio harveyi* is a pathogen that affects marine organisms (Austin and Zhang, 2006); it has been reported to cause the death of cage-cultured Asian catfish (Tendencia, 2002). The frequency of bacterial diseases affecting marine organisms has been increasing, and *V. harveyi* infections could cause severe economic losses in the aquaculture industry. Adhesion is the first step in bacterial infection, in which pathogenic bacteria adhere to the intestines or injured skin of the host, causing infection (Chen et al., 2008). Thus, inhibition of this step has gained interest among researchers (Kirk et al., 2010; Liu et al., 2013).

Bacteria can perceive gradients of environmental stimuli, including pH, temperature, osmolality, and the concentration of various chemicals. Active bacteria will move toward a favorable living environment (Colin and Sourjik, 2017). Chemotactic behavior allows bacteria to determine their course of action quickly and strategically in complex environments, which is essential for the induction of biofilm-related infections and pathogenic invasion into the host (Xue, 2015; Guo M. et al., 2017). Bacterial chemotaxis is a tightly regulated process, in which chemotactic signals are detected by methyl-accepting chemotaxis proteins (MCPs). MCPs can link to histidine protein kinases encoded by the *cheA* gene with the help of an adaptor protein encoded by *cheW*. CheV is a chemotactic connexin that can replace or enhance the function of *cheW* (Wadhams and Armitage, 2004). *CheA* and *cheY* are important constituents of a two-component system (Stock et al., 2000), the presence of repellents in the environment can stimulate the *cheA*, phosphorylated histidine kinase (CheA-P) phosphorylates CheB and CheY. The phosphorylated response regulator (CheY-P) increases its attraction to the motor protein *fliM* due to conformational changes, and the combination of the two makes the bacterial flagella movement clockwise (also called tumbling) to change direction stay away from repellents. On the contrary, the inducer in the environment can inhibit the autophosphorylation of *cheA*, and the response regulator (*cheY*) cannot be phosphorylated. The bacterial flagella move counterclockwise (also called swim), thereby tending to inducer. On the other hand, CheB-P and CheR change the methylation state of the receptor (MCPs) at a slower rate: CheR methylates it and CheB-P demethylates it. This methylation-demethylation cycle restores the activity of the associated CheA (Hess et al., 1988; Borkovich et al., 1989; Roman et al., 1992; Sockett et al., 1992). In *Salmonella enterica* (Haiko and Westerlund-Wikström, 2013) and *V. alginolyticus* (Huang et al., 2017), studies have confirmed that bacterial adhesion is controlled by chemotaxis-related genes.

Bacterial adhesion is a very complex process, and the ability of bacteria to adhere can be significantly affected by environmental factors (Yan et al., 2007). Studies have shown that bacterial adhesion is affected by physical and chemical factors such as temperature, salinity, ion concentration, and pH (Pianetti et al., 2012). The pH value affects the thickness of the electric double layer on the surface of pathogenic bacteria, thereby affecting the adhesion of bacteria to the surface of the substrate (Gorden et al., 1981). Different concentrations of monovalent ions have an effect on the adhesion of *Vibrio alginolyticus*, and the concentration of Na^+^ has the greatest effect on adhesion (Kogure et al., 1998). Na^+^ is the power source of *V. alginolyticus* polar flagella, thereby affecting its adhesion (Atsumi et al., 1992).

The process of adhesion of bacteria is connected to the movement of bacteria in response to a chemical stimulus. Chemical gradients are sensed through multiple transmembrane receptors, called methylaccepting chemotaxis proteins (MCPs), which vary in the molecules that they detect. These receptors may bind attractants or repellents directly or indirectly through interaction with proteins of the periplasmic space. The signals from these receptors are transmitted across the plasma membrane into the cytosol, where the two-component system is activated. The two-component system then induces tumbling by interacting with the flagellar switch protein FliM, inducing a change from counterclockwise to clockwise rotation of the flagellum. In the study of *V. alginolyticus*, the expression levels of ‘‘Bacterial chemotaxis’’ genes was consistent with the extent their adhesion decreased after the metal ion stress treatment (Kong et al., 2015). Subsequent studies also showed that chemotactic genes could affect the adhesion of *V. alginolyticus* (Wang et al., 2015).

Chemotactic genes are present in some bacteria, including *V. harveyi*. However, their roles in the adhesion of pathogenic bacteria still need to be identified. The aims of this research were as follows: (1) to identify chemotactic genes, which could potentially be associated with adhesion, (2) to determine the relationship between *V. harveyi* adhesion and *cheA*, *cheB*, *cheR*, *cheV*, and *cheY* activity, (3) to determine whether these genes participate in regulating adhesion under natural conditions, and (4) to detect the changes in the transcriptome of *V. harveyi* after silencing *cheA via* RNA interference (RNAi).

## Materials and Methods

### Bacterial Strain and Culture Conditions

*V. harveyi* (VH6110) was isolated from diseased *Larimichthys crocea.* The strain was identified to be pathogenic based on the regression of infection and was confirmed as *V. harveyi* by biochemical identification and 16S rRNA sequencing (Xu et al., 2010). The reference genome sequences were obtained through *de novo* assembly reference to near-source species. *V. harveyi* was cultivated in lysogeny broth at 28°C (LB; pH = 7, 2% NaCl, shaking at 200 rpm). *Escherichia coli* SM10 was purchased from TransGen Biotech (Beijing, China) and cultivated in LB broth or agar at 37°C. The pathogens and plasmids used in the study are presented in **Supplementary Table 1**.

To identify the chemotactic genes potentially associated with *V. harveyi* adhesion, the bacteria were subjected to stress with different concentrations of metal ions (Cu^2+^, Pb^2+^, Hg^2+^, or Zn^2+^) (Kong et al., 2015). *V. harveyi* grown in LB broth (pH = 7) was used as the control. Quantitative real-time polymerase chain reaction (qRT-PCR) was used to confirm gene expression in the adhesion-defective strain. All treatments were carried out using three independent replicates.

### Stable Gene Silencing

The methods used for stably silencing *V. harveyi* genes and treating *E. coli* SM10 have been reported previously (Darsigny et al., 2010; Huang et al., 2017), in which, pACYC184 vectors were *digested* using *Bam*HI* *and* Sph*I. Short hairpin (sh) RNA was obtained from Generay Biotech Co., Ltd. (Shanghai, China). The pACYC184 vectors were ligated using T4 DNA ligase (TaKaRa, Shiga, Japan). Recombinant plasmids were transformed into *E. coli* SM10 by heat-shock. Conjugation experiments were carried out by transferring the plasmids from *E. coli* SM10 to *V. harveyi.* An empty pACYC184 plasmid was transformed into *V. harveyi* as the control. LB medium containing chloramphenicol (34 μg/ml) and shRNA was used to select stably silenced *V. harveyi* cells (**Supplementary Table 2**).

### RNA Isolation

Total RNA was extracted using the TRIzol reagent (TransGen Biotech, Beijing, China). cDNA was synthesized using the TransScript^®^ ALL-in-One First-Strand cDNA Synthesis SuperMix and qPCR Assay Kit (TransGen Biotech, Beijing, China). The experiment was performed according to the manufacturer’s instructions.

### qRT-PCR

Gene silencing was confirmed by qRT-PCR (QuantStudio 6 Flex, Grand Island, NY, USA) using the SYBR Green qPCR Mix (Dongsheng Biotech, Guangdong, China). 16rRNA was used as the reference gene. All reactions were carried out in triplicate, and quantification was performed using the 2^−ΔΔCT^ method (Cikos et al., 2007). Primer sequences are listed in **Supplementary Table 3**.

### Mucus Preparation

Experiments on *L. crocea* were conducted in accordance with the specifications stated in the “Guide for the Care and Use of Laboratory Animals” published by the National Institutes of Health. Healthy *L. crocea* specimens were obtained from Fujian Fuding Seagull Fishing Food Co., Ltd. (Fujian, China). Mucus from the skin was collected according to a previously reported method (Huang et al., 2015). Briefly, *L. crocea* was washed with sterile PBS and the mucus from the skin was collected using a soft rubber spatula. Articulate material was removed by centrifuging twice (20,000 × *g*, 4°C, 30 min). The supernatant was passed through filters with pore sizes of 0.45 and 0.22 μm. The protein concentration was adjusted to 1 mg protein/ml using sterile PBS (Bradford, 1976).

### Adhesion Assay

The experiment on bacterial adhesion was performed as described previously (Huang et al., 2015). Briefly, 20 μl of mucus from *L. crocea* was applied to a glass slide (22 mm × 22 mm) and fixed with methanol at 28°C for 20 min. Then, 200 μl of the suspension of *V. harveyi* adjusted to an OD_600_ of 0.3 (concentration of 3.0 × 10^8^ CFU/ml) was applied evenly on the mucus-coated glass slides. The slides were incubated at 28°C for 2 h and washed thrice with PBS. The *V. harveyi* cells were fixed with 4% methanol for 30 min and stained with 1% crystal violet for 3 min. The slides were examined under a light microscope at *1,000*× magnification. The number of *V. harveyi* cells was counted in 20 sections. The experiment was carried out using *positive controls with V. harveyi only* and *negative controls* with sterile PBS.

### In Vitro Biofilm Assay

*A 12 h-old* c*ulture of V. harveyi* was resuspended in sterile PBS, and its OD_600_ was adjusted to 0.2 (2.0 × 10^8^ CFU/ml) using sterile LB. Then, 200 μl of this suspension was added to the wells of a 96-well plate. Sterile LB was used in the blank control group. The plates were incubated at 28°C for 24 h. The plate contents were shaken and washed thrice using 200 μl of sterile PBS to remove the non-adherent bacteria. The microtiter plates were dried at 60°C for 10 min, and 125 μl of a 0.1% crystal violet solution (Merck KGaA, Darmstadt, Germany) was added to each well; the plates were then incubated for 10 min. The microtiter plates were washed thrice using sterile PBS. Then, the biofilms were solubilized using 33% acetic acid, and OD_590_ was measured using a microtiter plate reader (Bio-Rad, Hercules, CA, USA). Each experiment was performed in *triplicate*.

### Soft Agar Plate Motility Assay

Bacterial motility was determined using a *soft agar* plate *according to* a method described previously (Luo et al., 2016). The OD_600_ of bacterial cultures grown overnight in LB was adjusted to 0.3, and plates of LB agar (0.3% agar) were seeded using 1 μl of the culture suspension. After incubating the *plates* at 28°C for 24 h, the diameters of the bacterial colonies were measured. Each experiment was performed in triplicate.

### Capillary Assay

Bacterial chemotaxis was tested using a method reported previously (Huang et al., 2017). A capillary tube with an *inside diameter* of *0.1* mm *was filled with* mucus, leaving one end open. Then, the tube was filled with 2.5 ml of bacterial suspension (1.0 × 10^9^ CFU/ml), the open end of the capillary is placed in the bacterial solution. After incubation for 1 h at 28°C, the LB agar plates were inoculated with the contents of the tube to accurately determine the number of bacteria present in the capillary tube. Bacterial chemotaxis was determined by comparing the number of bacteria in the tube with that of the negative control, which consisted of a capillary filled with mucus-free buffer. Samples were tested in triplicate in each group.

### Transcriptomic Analysis

#### Library Preparation and Sequencing

Total RNA was isolated and purified from the bacterial solution (sample collected in triplicate), and the concentration was measured using the Nanodrop 2000. The RNA integrity number (RIN) was measured using an Agilent 2100 Bioanalyzer System. The TruSeq™ RNA sample preparation Kit (Illumina, San Diego, CA, USA) was used to construct the rRNA-depleted and RNA-fragmented libraries. Here, dUTP was used for construction of the second strand of cDNA, which resulted in the presence of A/U/C/G in the new strand. End repair and *adapter ligation led to the* adenylation of the 3’ ends. Then, the UNG enzyme was used to digest the second strand of cDNA; thus, the first strand only was included in the libraries. Transcriptome sequencing (2 × 150 bp, paired-ended) was performed using an Illumina Hiseq from Majorbio Biotech Co., Ltd. (Shanghai, China).

#### Data Analysis

The original sequencing data was filtered to obtain clean data using Sickle and SeqPrep. Based on the method of Burrows-Wheeler, high-quality sequence data were compared to those of the reference genome obtained from NCBI (NZ_CP009467.1). EdgeR (http://www.bioconductor.org/packages/2.12/bioc/html/edgeR.html) was used to detect differentially expressed genes (DEGs) between the two samples with significant false discovery rate (FDR) *P* value < 0.05 and |log2FC| ≥ 1, where FC = fold change.

The H-cluster method was used for cluster analysis to determine the expression patterns of DEGs. All DEGs and gene ontology (GO) terms were mapped to a reference database (http://www.geneontology.org/) to indicate gene function in the samples. Enrichment analysis of DEGs in the Kyoto Encyclopedia of Genes and Genomes (KEGG) pathway was conducted using the KOBAS software, and Fisher’s exact test was used for calculations. The Benjamini-Hochberg Procedure (FDR) was used to analyze for KEGG pathway *enrichment. P* < 0.05 was considered statistically significant. The data of significantly expressed genes was validated using qRT-PCR. Genes and primer sequences are listed in **Supplementary Table 4**.

### Environmental Impact on Adhesion Ability

To investigate the effects of different temperatures, *V. harveyi* was cultured in LB broth (supplemented with 2% NaCl, pH = 7) at 4, 15, 28, 37, and 44°C. To investigate the effects of different pH levels, *V. harveyi* was cultured in LB broth (supplemented with 2% NaCl) adjusted to a pH of 5, 6, 7, 8, or 9 at 28°C. To investigate the effects of different salinities, *V. harveyi* was cultured in LB broth (pH = 7) with 0.8, 1.5, 2.5, 3.5, or 4.5% NaCl at 28°C. Each treatment consisted of six independent replicates. After harvesting and re-suspending, the adhesion ability of *V. harveyi* was measured, RNA was extracted from the bacteria, and reverse transcription were performed, expression levels of *cheA*, *cheB*, *cheR*, *cheV*, and *cheY* were determined using qRT-PCR according to the method described in *qRT-PCR*.

### Data Processing

The data are summarized as the mean ± standard deviation. The data was analyzed by *one*-*way ANOVA* using 17.0 Statistics (Chicago, IL, USA). *P* < 0.05 was considered statistically significant.

## Results

### Adhesion and Gene Expression of *Vibrio harveyi* Under Stress

**Figure 1:**
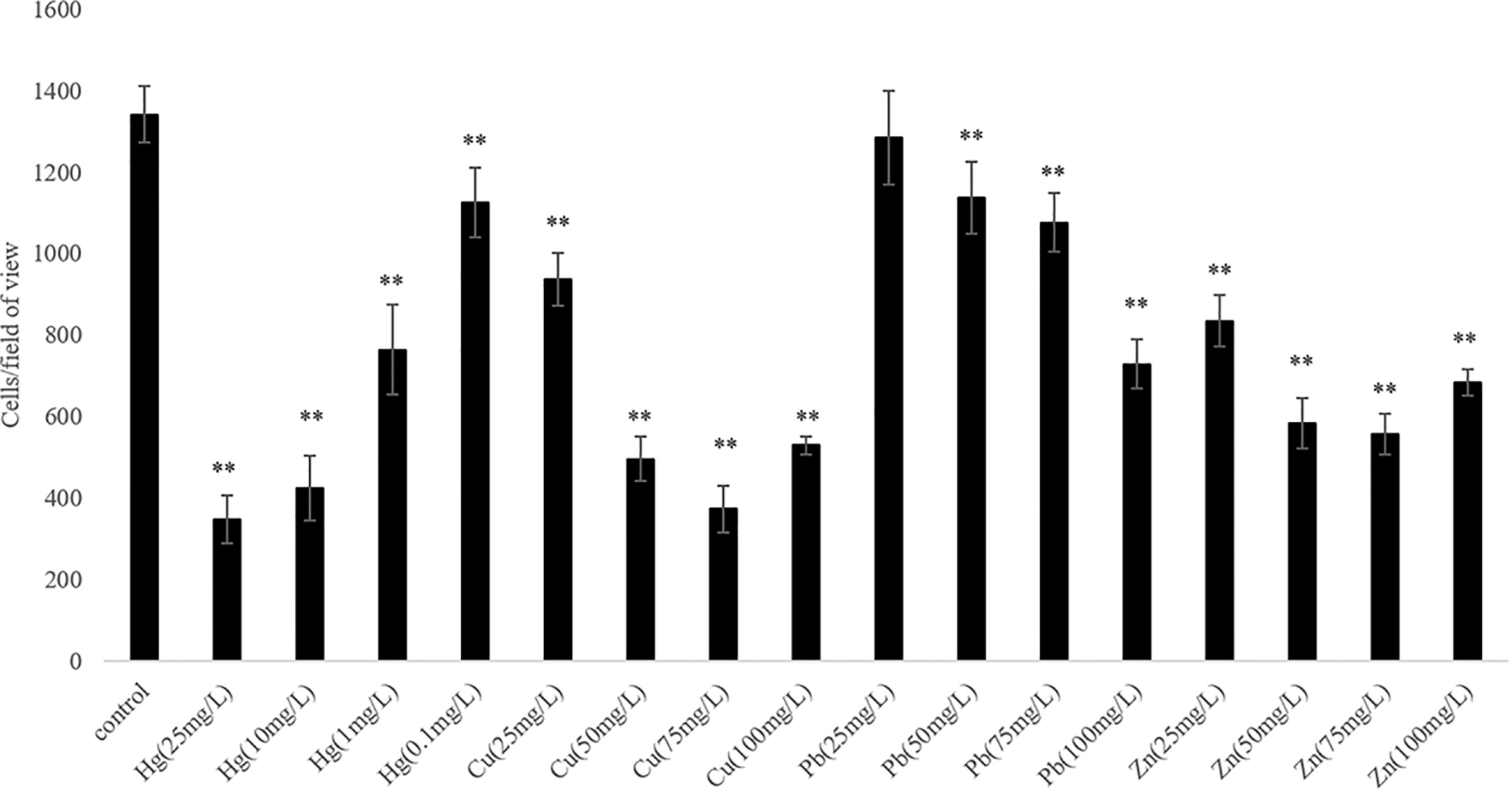
The adhesion abilities of the wild-type and stressed *Vibrio harveyi* cells. Data are presented as the mean ± SD; experiments were performed on three independent replicates per group. **P < 0.01 compared to the control.

**Figure 2:**
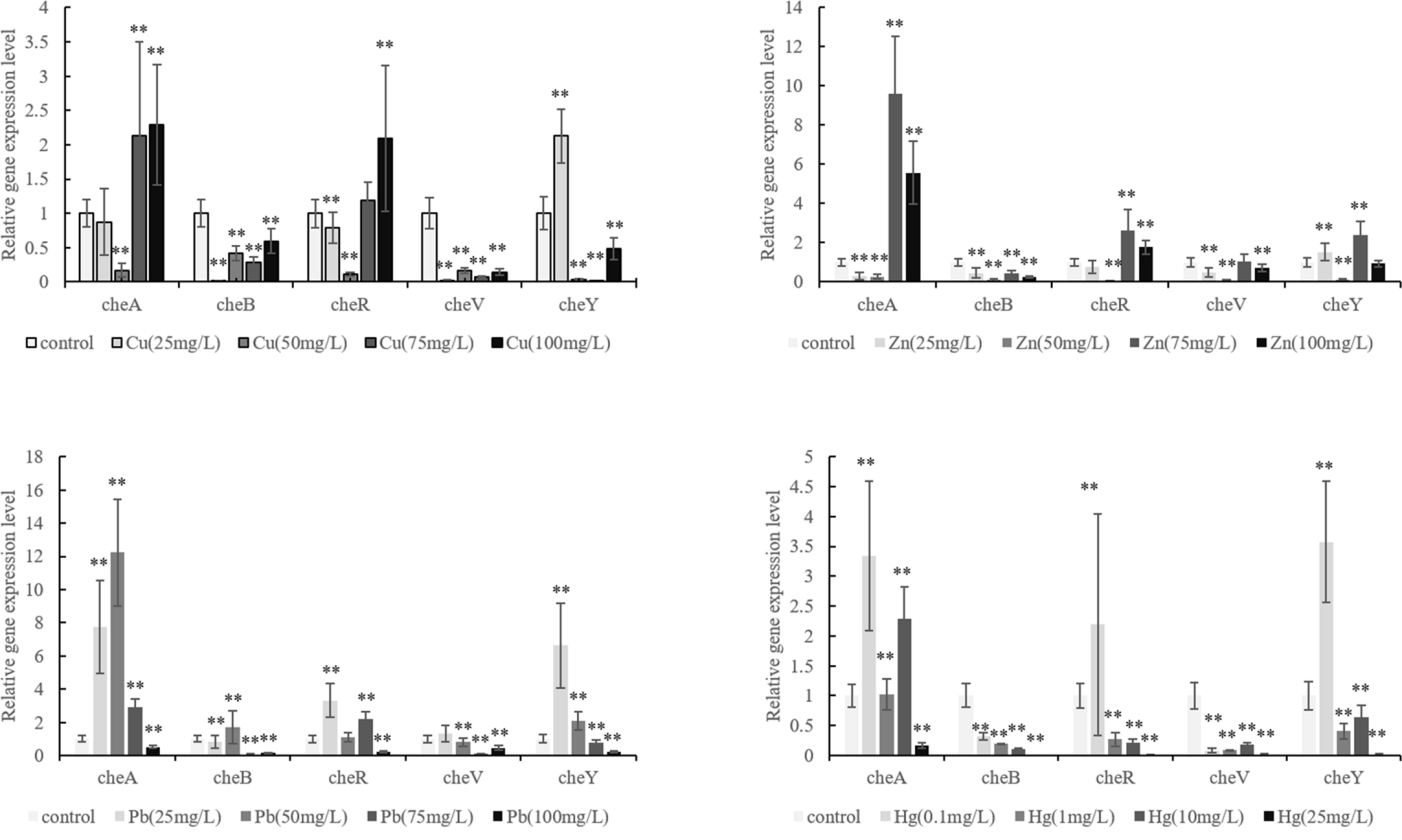
Expression of *cheA*, *cheB*, *cheR*, *cheV*, and *cheY* in the control and stressed *Vibrio harveyi* cells confirmed *via* qRT-PCR. Data are presented as the mean ± SD; experiments were performed on three independent replicates per group. **P < 0.01 compared the control.

We observed that the ability of *V. harveyi* to adhere to crocea mucus changed significantly after the bacteria were subjected to stress, and most of the cases were decreased (**Figure 1**, *P* < 0.01). In addition, the results of qRT- PCR showed that chemotactic genes were very sensitive to metal ions, most of the times, treatment with Cu^2+^, Hg^2+^, Zn^2+^, and Pb^2+^ significantly reduced the expression of *cheA*, *cheB*, *cheR*, *cheV*, and *cheY*. Hg^2+^ had the greatest effect on adhesion and gene expression. In particular, the expression of *chaA* gene was significantly upregulated most frequently after ion stress (**Figure 2**, *P* < 0.01). The adhesion ability of *V. harveyi* may be regulated by chemotactic genes, but the regulation mechanism is complex.

### Effects of Stable Gene Silencing

**Figure 3:**
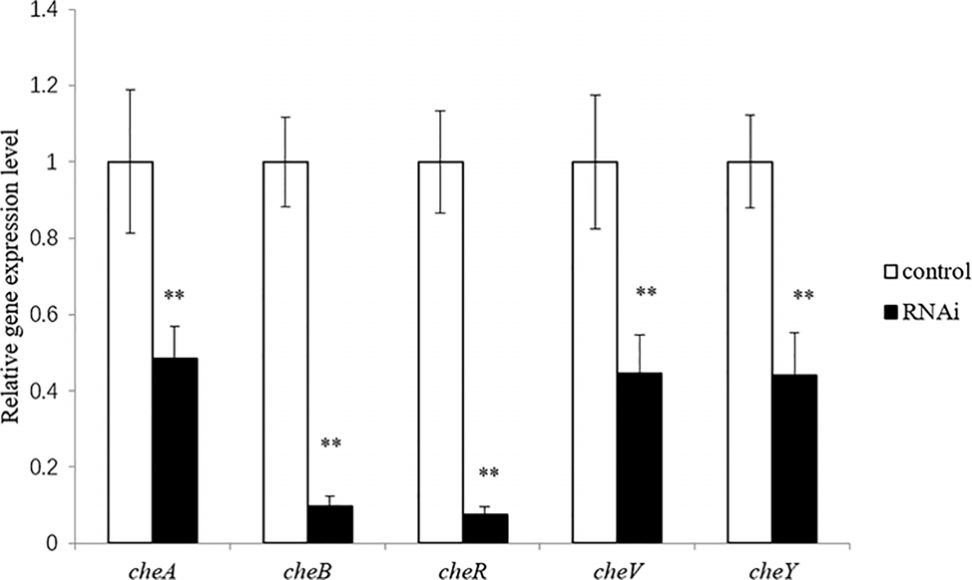
Expression of *cheA*, *cheB*, *cheR*, *cheV*, and *cheY* in the control and RNAi-silenced *Vibrio harveyi* cells confirmed *via* qRT-PCR. Data are presented as the mean ± SD; experiments were performed on three independent replicates per group. **P < 0.01 compared to the control.

**Figure 4:**
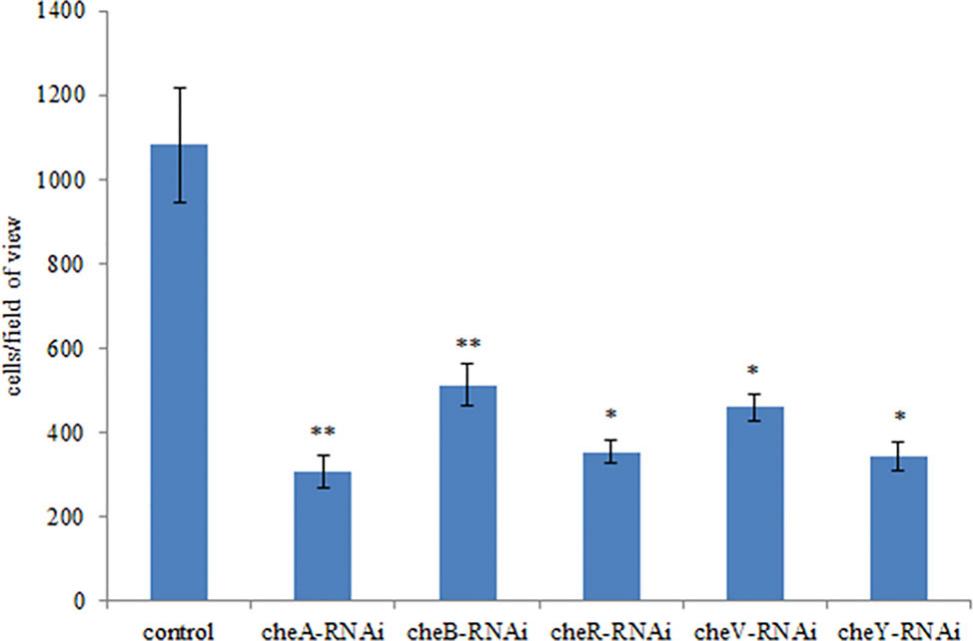
Adhesion abilities of the control and stably silenced *Vibrio harveyi* cells. Data are presented as the mean ± SD; experiments were performed on three independent replicates per group. **P < 0.01, *P < 0.05 compared to the control.

The correlation between genes and adhesion was confirmed through RNAi and adhesion experiments. Expression of the stably silenced *cheA*, *cheB*, *cheR*, *cheV*, and *cheY* genes decreased by 2.1-, 10.4-, 13.4-, 2.2, and 2.3-fold, respectively, compared to the control gene (**Figure 3**, *P* < 0.01). The ability of the clones with silenced genes to adhere to mucus was significantly reduced. The number of adherent *V. harveyi* colonies in the control group was 1,081 ± 112 cells/field. The number of adherent *V. harveyi* colonies in the *cheA-*, *cheB-*, *cheR-*, *cheV-*, and *cheY*-RNAi groups was 309 ± 38, 512 ± 50, 354 ± 26, 460 ± 32, and 344 ± 35 cells/field, respectively (**Figure 4**, *P* < 0.05). These results suggest that bacterial adhesion could be reduced by stably silencing *cheA*, *cheB*, *cheR*, *cheV*, and *cheY*.

**Figure 5:**
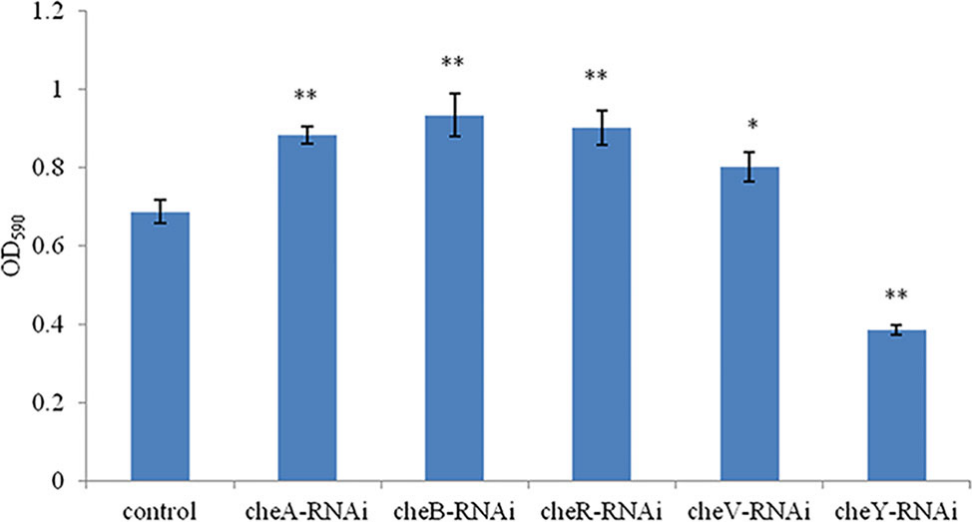
The ability of the control and stably silenced *Vibrio harveyi* cells to form biofilms in lysogeny broth (LB) medium at 28°C. Data are presented as the mean ± SD; experiments were performed on three independent replicates per group. **P < 0.01, *P < 0.05 compared to the control.

A comparison between the abilities of the control *V. harveyi* and the stably silenced strains to form bacterial biofilms is presented in **Figure 5**. Compared to the control strain, the strains silenced with *cheA*-, *cheB*-, *cheR*-, and *cheV*-RNAi demonstrated an increased ability to form biofilms after 24-h incubation. This phenomenon was not observed in *cheY*-RNAi strains. The *cheY* gene silenced *V. harveyi* showed a reduced ability to form biofilms, this phenomenon needs further explanation.

**Figure 6:**
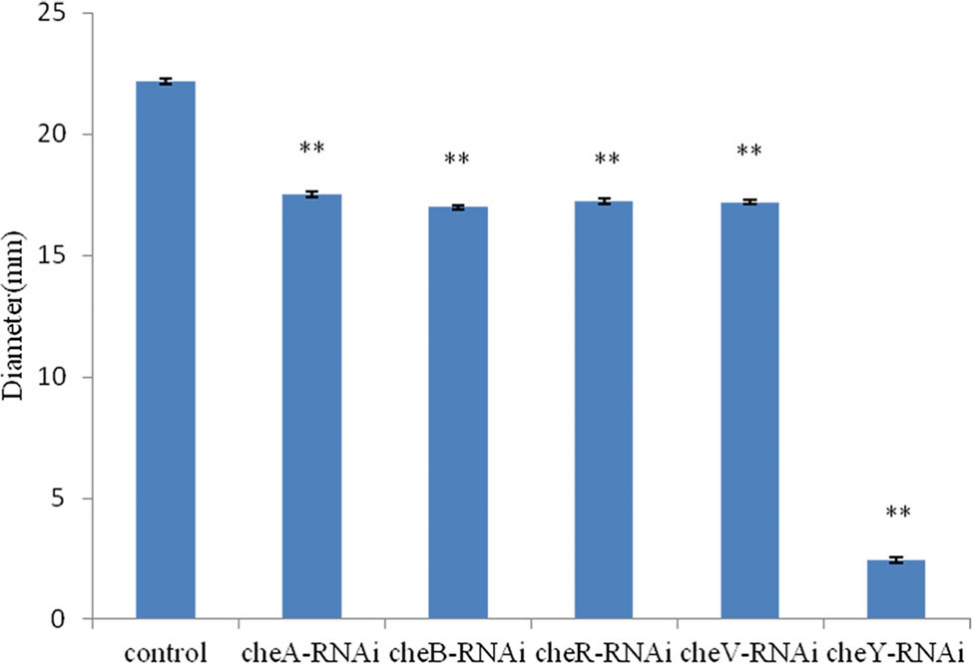
Motility of *Vibrio harveyi* on soft agar plates. Data are presented as the mean ± SD; experiments were performed on three independent replicates per group. **P < 0.01 compared to the control.

The bacterial motility of stably silenced strains was significantly reduced, and the *cheY*-RNAi bacteria was the least motile (**Figure 6**). Typical images of the spreading of stably silenced *V. harveyi* strains and control were showed in **Supplementary Figure 1**. Interestingly, we found that *cheY* gene-silencing strains always show more special phenomena than other silent strains.

**Figure 7:**
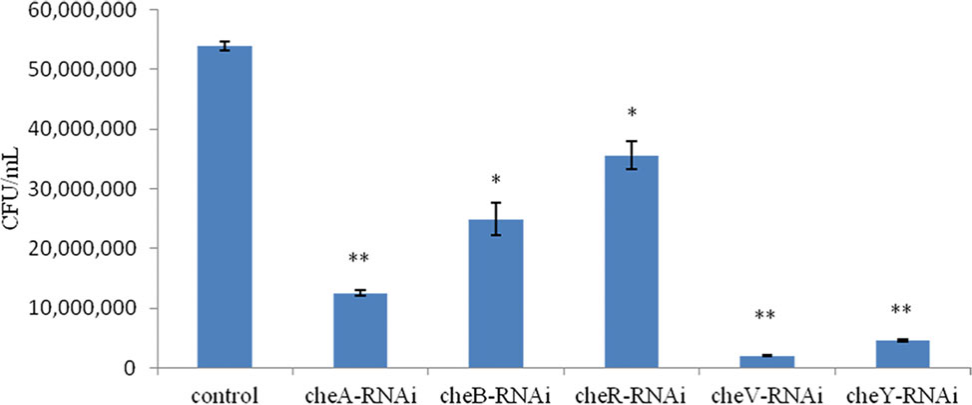
Chemotactic ability of stably silenced *Vibrio harveyi* strains and the control to move toward the mucus. Data are presented as the mean ± SD (n = 3). **P < 0.01, *P < 0.05 compared to the control.

We observed that the extent of chemotaxis toward the skin mucus of *L. crocea* was higher in the control *V. harveyi*. Stable silencing of the genes significantly reduced the chemotactic ability of the bacteria. The chemotactic ability decreased by 4.2-fold (*cheA-*RNAi), 2.1-fold (*cheB-*RNAi), 1.5-fold (*cheR-*RNAi), 26.7-fold (*cheV-*RNAi), and 11.7-fold (*cheY-*RNAi), compared to the control *V. harveyi* strain (**Figure 7**, *P* < 0.05). The *cheY* gene silenced strain did not show the lowest chemotaxis ability, but the *cheV* silenced strain. The biofilm formation ability, motility, and chemotaxis of wild *V. harveyi* strains under stress by Cu^2+^ (50mg/L) and Zn^2+^ (50mg/L) were showed a certain degree of reduction, proved that these assays were working properly and with expected dynamic ranges (**Supplementary Figure 2**).

**Figure 8:**
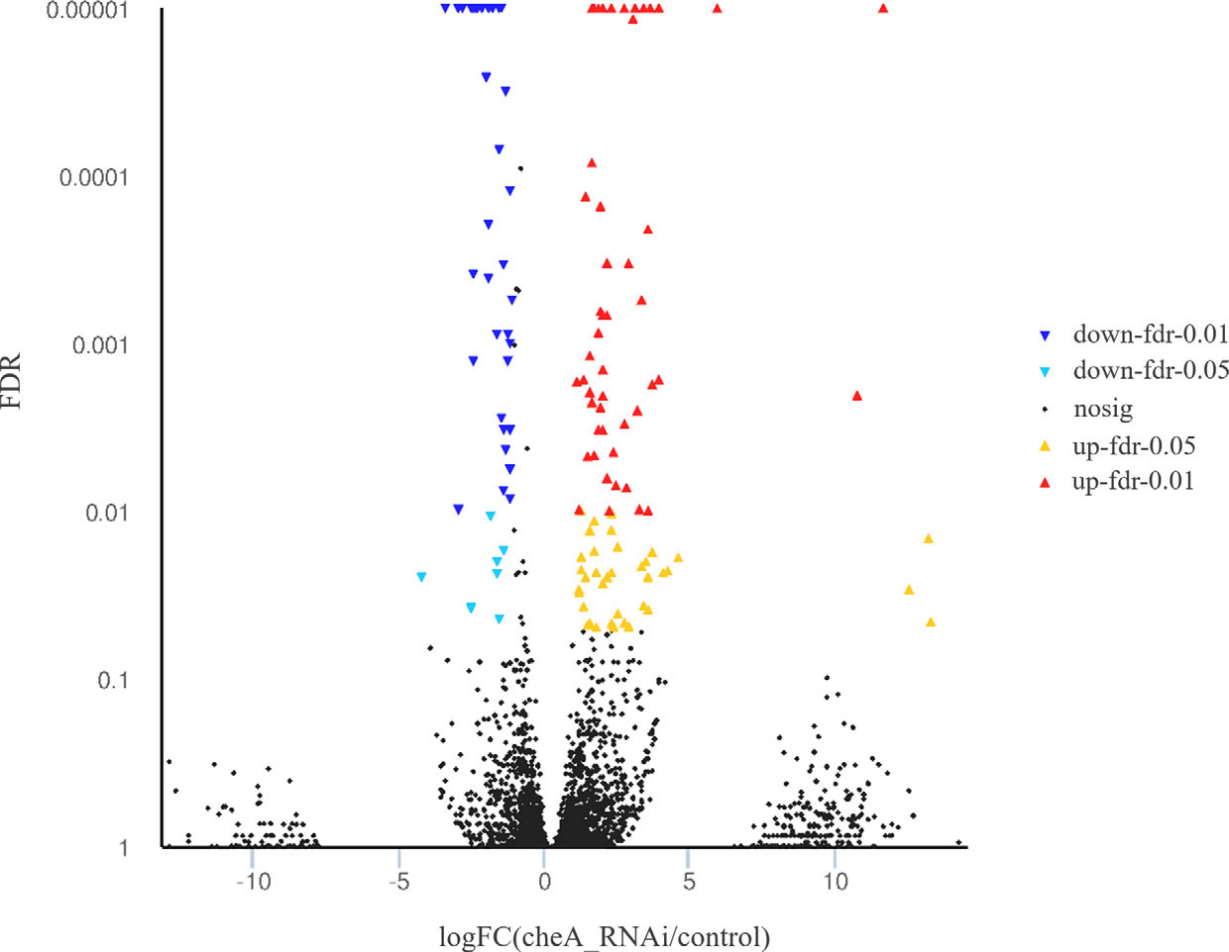
Volcano plot of all genes. X and y axes represent the fold change values of the *cheA*-RNAi strain/control strain and statistical test value [false discovery rate (FDR)], respectively. Higher values represent more significant differences. Each dot represents one gene. Red and blue dots indicate significantly upregulated and downregulated genes, respectively. Black dots represent genes with expression changes that are not significant.

The *cheA* gene is located at the core of the bacterial chemotactic system, and *cheA* gene silenced strains show the lowest adhesion ability, thus, RNA sequencing libraries were constructed using the control *V. harveyi* and *cheA*-RNAi strains. Quality control on raw Illumina reads provided high-quality reads, which were mapped to the *V. harveyi* reference genome. The mapping rate was 89.63 and 89.73% for the control *V. harveyi* and *cheA*-RNAi strains, respectively. EdgeR was used to calculate the DEGs between the two samples, and 5,348 genes were identified from the *cheA*-RNAi strains. Compared to the control, 134 genes were significantly differentially expressed in the *cheA*-RNAi cells. There were 45 downregulated and 89 upregulated genes (**Figure 8**).

The detected differentially expressed genes of *cheA*-RNAi strains were annotated with GO function to clarify gene function, among which 89 significantly upregulated genes were mapped to 27 GO terms and 45 significantly downregulated genes were mapped to 20 GO terms (**Supplementary Figure 3**). These significantly upregulated genes are mainly involved in functions such as biological regulation, cellular processes, metabolic processes, regulation of biological processes, formation of macromolecular complexes, binding, catalytic activities, localization, and establishment of localization.

**Figure 9:**
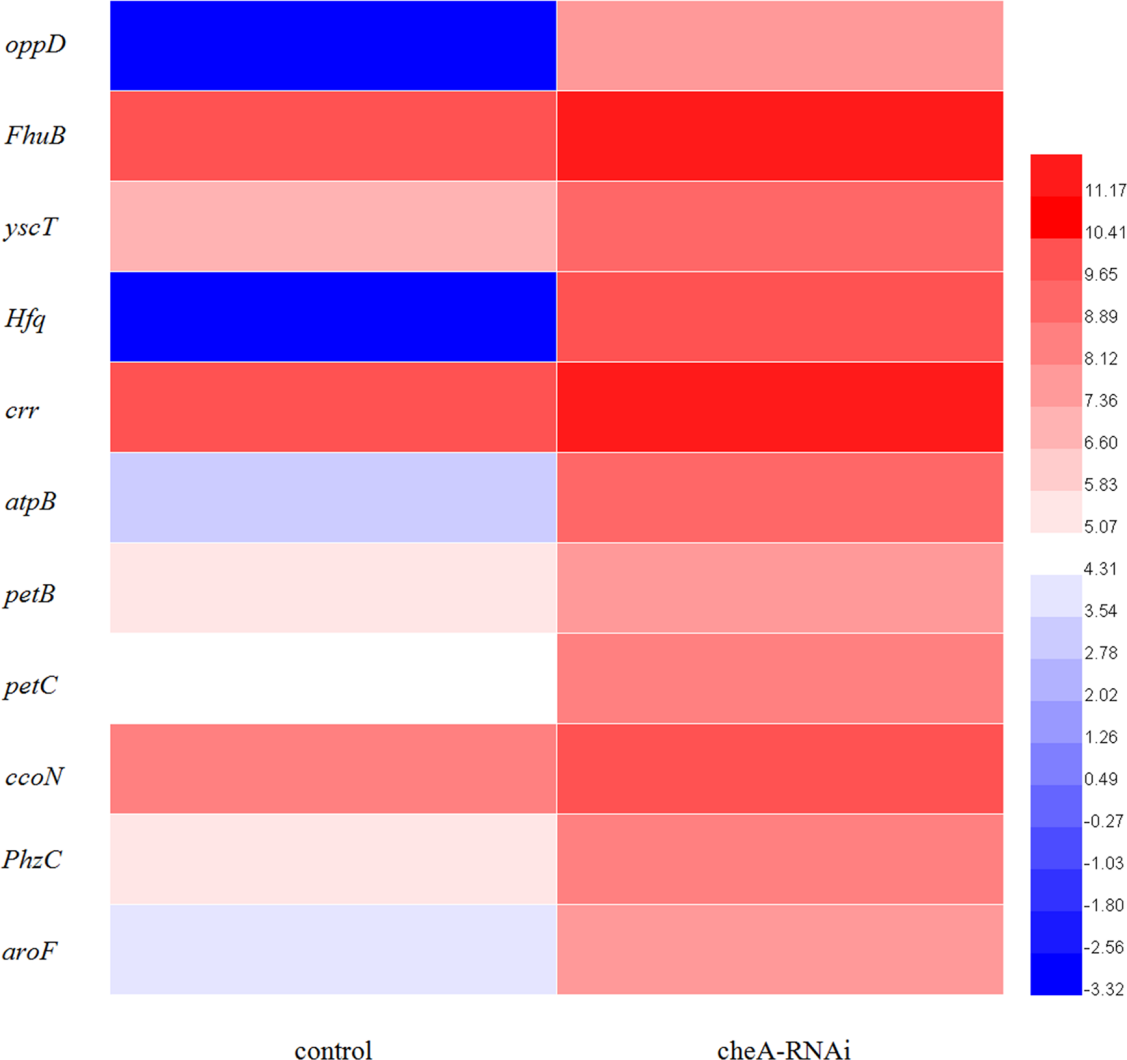
Heat map of the DEGs involved in adhesion-related pathways (adjusted FDR < 0.05; |log2FC| ≥ 1; three replicates). Values represent log2-fold change. Colors of the log-transformed transcripts represent the mean FPKM values. Blue and red indicate decreased and increased expression, respectively.

**Figure 10:**
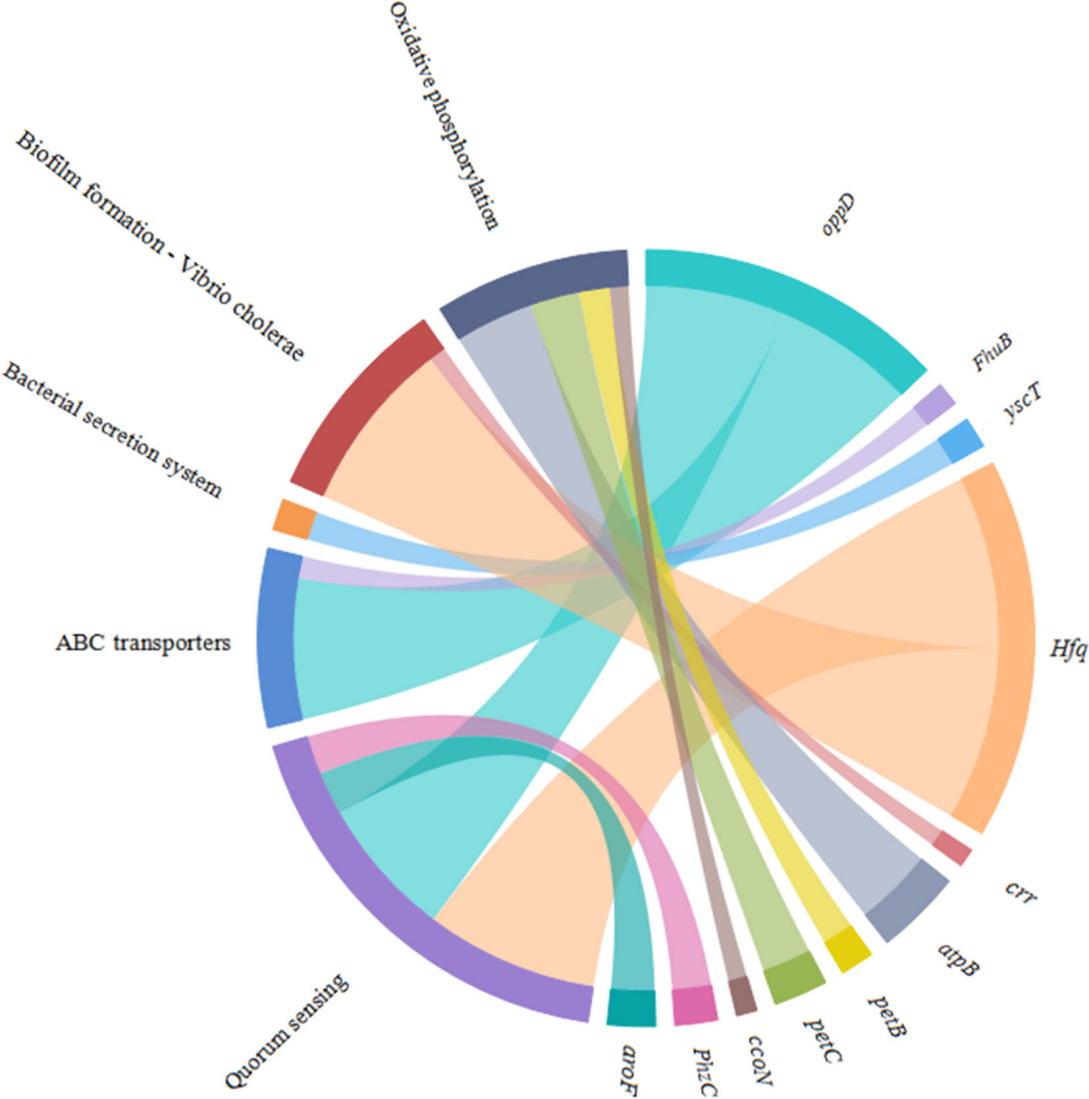
Chordal graph of the DEGs to KEGG pathways. The width of the chordal represents the log2FC value.

According to the KEGG database, DEGs in *V. harveyi* were mapped to 62 KEGG pathways. The largest number of pathways mapped included those for the “biosynthesis of amino acids,” “metabolic pathways,” “biosynthesis of secondary metabolites,” “two-component system,” “bacterial secretion system,” and “ABC transporters,” among others. Among all the KEGG pathways, “biofilm formation-*Vibrio cholerae*,” “ABC transporters,” “bacterial secretion system,” “oxidative phosphorylation,” and “quorum sensing” were associated with bacterial adhesion (Kolenbrander et al., 1998; Watnick and Kolter, 1999; Paola et al., 2003; Koutsoudis et al., 2006; Guo L. et al., 2017). Upon analyzing the expression of the DEGs involved in these pathways, we determined that the expression of many of the genes in these pathways was significantly upregulated in *cheA*-RNAi cells (**Figure 9**). The relationships between genes and KEGG pathways are presented in **Figure 10**. We used qRT-PCR to experimentally confirm DEG expression changes and validate the RNA-seq results (**Supplementary Figure 4**). qRT-PCR yielded similar expression patterns, which supports the reliability and accuracy of the RNA-seq data.

### Effects of Different Environmental Conditions

**Figure 11:**
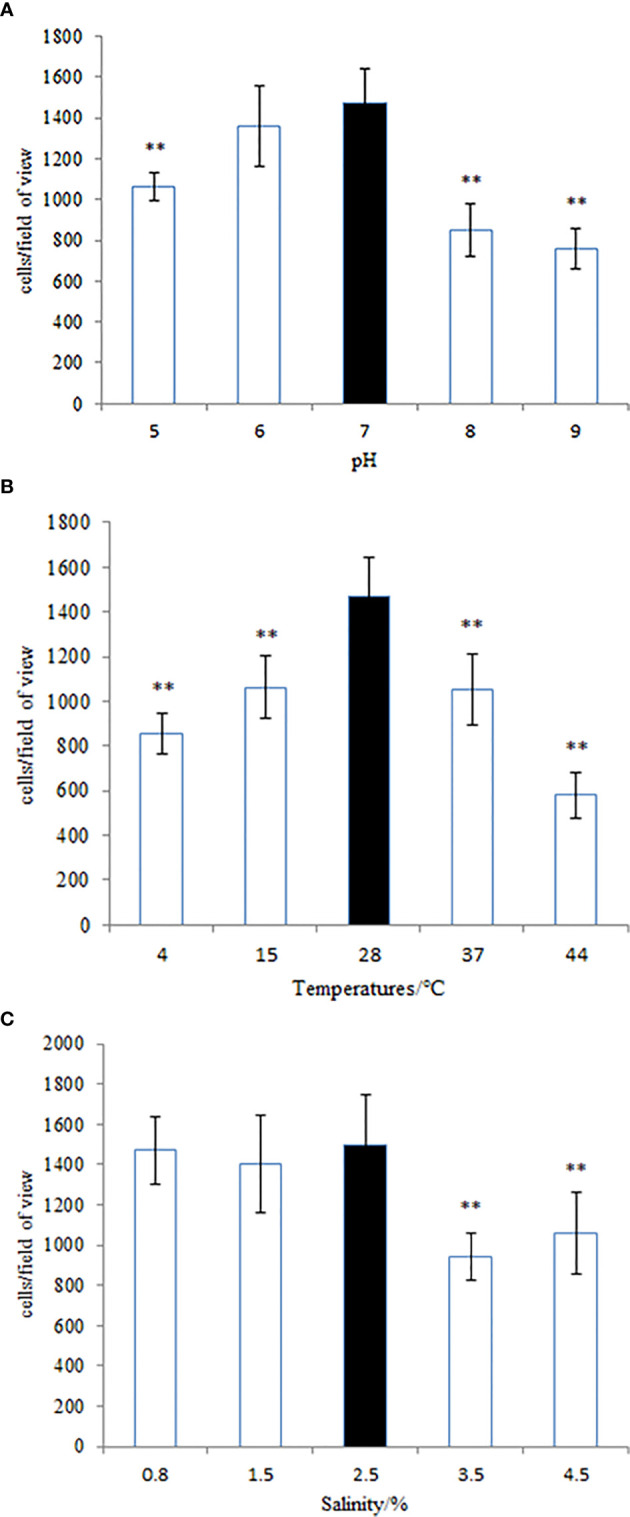
The adhesion ability of the wild-type *Vibrio harveyi* strain under different conditions of **(A)** pH, **(B)** temperature, and **(C)** salinity. Data are presented as the mean ± SD, and each treatment was performed on six independent replicates. **P < 0.01 compared to the control.

Many pathogenic bacteria can induce an adaptable response to environmental stimuli, so studying the influence of environmental factors on the gene expression will help to understand the molecular mechanism of the adhesion of *V. harveyi* in the environment. The adhesion ability of *V. harveyi* under different environmental conditions is presented in **Figure 11**. The adhesion of *V. harveyi* to mucus was found to be stronger under acidic and neutral environments than under alkaline conditions (**Figure 11A**). Moreover, the adhesion of *V. harveyi* was reduced at both high and low temperatures (**Figure 11B**). The adhesion ability of *V. harveyi* also was reduced under conditions of high salinity; however, no significant difference was observed with 0.8, 1.5, and 2.5% salinity (**Figure 11C**). The environmental conditions significantly affected the expression of all five genes. The expression of *cheA* and *cheR* increased significantly under acidic and alkaline conditions, whereas the expression of the other three genes was the highest at pH = 7 (**Supplementary Figure 5**). Gene expression decreased at low temperatures, whereas *cheV* expression increased at 37 and 44°C and *cheA* expression increased at 44°C. *cheR* expression was the most stable over the different temperature conditions (**Supplementary Figure 6**). The lowest expression of *cheA*, *cheB*, *cheR*, *cheV*, and *cheY* was observed under 3.5% salinity (**Supplementary Figure 7**). These data indicate that *cheA*, *cheB*, *cheR*, *cheV*, and *cheY* may participate in regulation of adhesion in the natural environment.

## Discussion

*V. harveyi* is an important pathogen (Yang and Defoirdt, 2015), and studies on *V. harveyi* have mainly focused on quorum sensing (Yang and Defoirdt, 2015; Van Kessel et al., 2018; McRose et al., 2018). Bacterial adhesion is a virulence factor and leads to infection through the attachment of bacteria to the surfaces of host membranes (Hamed et al., 2018). Understanding the mucus adhesion mechanisms of pathogens will aid in the prevention of bacterial diseases. Bacterial adhesion is regulated by known genes, such as *CdiA* gene, flagellum genes (*flrA*, *flrB*, and *flrC*) and global regulator RpoN and GacS, which have been reported in *E. coli* (Ruhe et al., 2015), *V. alginolyticus* (Luo et al., 2016), and *Pseudomonas aeruginosa* (Duque et al., 2013). However, it has not been reported in *V. harveyi*. The detailed adhesion mechanisms have not yet been fully elucidated.

Using RNA-seq, Huang et al. (2017) and Kong et al. (2015) confirmed that a relationship exists between adhesion and the bacterial chemotaxis pathway. In our study, the adhesion ability of *V. harveyi* was significantly decreased upon treatment with Cu^2+^, Pb^2+^, Hg^2+^, and Zn^2+^. Additionally, results of qRT-PCR demonstrated that the five genes responsible for chemotactic activity (*cheA*, *cheB*, *cheR*, *cheV*, and *cheY*) had been significantly changed in adhesion-deficient strains. Bacterial adhesion of *V. harveyi* was reduced upon RNAi-mediated gene silencing (**Figure 4**), thus indicating that *cheA*, *cheB*, *cheR*, *cheV*, and *cheY* may play important roles in this process.

We observed that the chemotaxis and motility of *cheA*-, *cheB*-, *cheR*-, *cheV*-, and *cheY*-RNAi cells had significantly decreased. Chemotaxis is the activity of bacteria that involves movement away from the surface of the zooplankton toward the mucus; however, it is not directly related to adhesion (Bordas et al., 1998). The chemotactic system plays a vital role in inducing motility (Burkart et al., 1998). The chemotaxis system integrates the signals from external and internal sensors through a signal transduction cascade consisting of MCPs, CheW/CheV, CheA and CheY, while other factors such as CheB, CheR, CheC, CheZ, or CheX may modulate and fine-tune the signal transduction cascade, with diverse combinations observed throughout the bacterial kingdom. It is known that *cheA* and *cheB* are putatively involved in pilus-mediated twitching motility (Sonnenschein et al., 2012). *cheV* encodes a chemotactic connexin that can affect the ATPase activity of CheA. The CheY protein is the final effector protein in the signal transduction cascade, directly interacting with the flagellar switch. The observation that lowest mobility of *cheY*-RNAi supports this theory. Interestingly, we observed that the ability of *cheA*-, *cheB*-, *cheR*-, and *cheV*-RNAi cells to form biofilms had increased, whereas that of *cheY*-RNAi had decreased. This result is similar to that described previously, suggesting that, although bacterial adhesion and biofilm formation are complex multi-step processes causing diseases, they are not directly related, biofilm formation is not dependent on the extent of initial adherence of bacteria to the substrate. Biofilm formation is more likely to be dependent on cell-to-cell adhesion rather than on the amount of cells initially attached to the surface. Adherence is a complex phenomenon involving a variety of surface factors on the bacterium (Cerca et al., 2005). Previous studies have implicated chemotaxis sensors were involved in biofilm formation. Tlp3 (Cj1564) mutants showed increased biofilm formation (Rahman et al., 2014), while CetZ (Tlp8) mutants showed decreased biofilm formation (Chandrashekhar et al., 2015). Defect in motility reduces the opportunities for bacteria to come into contact with surfaces, and the deletion of *plzB* in wild-type *V. cholerae* results in a decrease in biofilm formation and motility (Pratt et al., 2007). Thus, a serious decline in the motility of *cheY*-RNAi may decrease the extent of biofilm formation. Bacterial adhesion is severely affected by motility (Beier and Gross, 2006), the result shows that motility is one of the approaches through which chemotactic genes influence adhesion, however, further research is required to fully understand these processes.

The loss of a single gene can significantly alter the transcriptional landscape of a bacterium (Richmond et al., 2016). In our study, silencing *cheA* significantly altered the transcriptome of *V. harveyi*. GO analysis revealed that many DEGs were involved in localization and the response to stimuli, which were relevant to bacterial adhesion (Kong et al., 2015). Changes in genes in the KEGG pathway related to adhesion (“biofilm formation-*Vibrio cholerae*,” “ABC transporters,” “oxidative phosphorylation,” “quorum sensing,” and “bacterial secretion system”) prompt us *V. harveyi* may regulate the expression of these genes to cope with the decreased adhesion ability caused by *cheA* silencing.

The process of bacterial adhesion is linked to the movement of bacteria in response to chemical stimuli, which depends on bacterial chemotaxis. In this process, the bacteria sense signals transmitted to the cytosol, and the two-component system is activated. The two-component system interacts with the bacterial flagella and affects its adhesion (Benhamed et al., 2014). Additionally, the process of bacterial adhesion is affected by adhesins (Haiko and Westerlund-Wikström, 2013). Thus, a “bacterial secretion system” could mediate adhesion by controlling the secretion of intercellular polysaccharide adhesins. The results indicated that the DEGs identified in this study could affect bacterial adhesion. It is likely that *V. harveyi* could adapt to the reduced stimulus for adhesion caused by *cheA* silencing by regulating adhesion-related pathways. In the process of bacterial adhesion, cheA gene may affect adhesion by regulating bacterial response to environmental stimuli, which needs to be further confirmed.

The adhesion capacity of bacteria was dependent on environmental factors, particularly salinity and temperature (Benhamed et al., 2014), and the RNA-seq results suggest that the regulation of chemotactic genes on adhesion may be related to environmental stimuli. Therefore, we studied the adhesion ability and gene expression of *V. harveyi* in different environments. At different temperatures, an inverted U-shaped trend was observed in the adhesion ability of *V. harveyi*, with the highest adhesion occurring at 28°C, which is the same as that observed for *V. alginolyticus* (Huang et al., 2016). The expression of both *cheA* and *cheV* increased at high temperatures, whereas that of *cheR* was minimal under different temperature conditions. The expression of *cheB* and *cheY* was highly downregulated under both high and low temperature conditions. In summary, *cheB* and *cheY* could be the key regulatory genes responsible for the adhesion of the bacterium at different temperatures. Additionally, expression of these genes was highly impacted by low temperatures, potentially owing to a decrease in the metabolic levels of *V. harveyi* under these conditions. No similar trends in the expression of the five genes and the adhesion ability of *V. harveyi* under varying salinities were observed. *cheR* is essential for stable adhesion of *V. harveyi* at salinities of 1.5 and 2.5%, and further research is needed to explain the regularity of the effect of salinity on the adhesion of *V. harveyi*. A higher adhesion ability of *Vibrio anguillarum* (Balebona et al., 1995) and *V. alginolyticus* (Yan et al., 2007) was observed in acidic environments, and this result was similar to that observed with *V. harveyi*. In this study, under acidic conditions, the expression of *cheA* was the highest. However, the lowest expression of *cheA* was observed under neutral conditions, indicating that it has no obvious correlation with the adhesion ability of the bacteria. The results showed that the expression of *cheB*, *cheV*, and *cheY* showed trends similar to the bacterial adhesion ability, suggesting that their expression may be linked to the regulation of bacterial adhesion in a variable pH environment. Although the expression of *cheR* was upregulated to suit different pH conditions, no significant effect on adhesion was observed. This indicates that environmental conditions can affect bacterial adhesion and the expression of chemotactic genes, but how the genes regulate the adhesion of bacteria in a complex environment requires further research.

## Conclusion

In conclusion, our study revealed the following results: (1) The chemotactic genes *cheA*, *cheB*, *cheR*, *cheV*, and *cheY* may regulate the adhesion ability of *V. harveyi* by affecting bacterial motility, and participate in the regulation of adhesion at different temperatures, salinities, and pH values; (2) RNAi-mediated *cheA* silencing altered the transcriptional landscape of *V. harveyi* and regulated the expression of genes involved in adhesion-related pathways. Understanding the relationship between the expression of *cheA*, *cheB*, *cheR*, *cheV*, and *cheY* and bacterial adhesion will provide insights on the mechanisms by which pathogens adhere to the mucus and assist in uncovering new avenues to prevent bacterial diseases.

## Data Availability Statement

The datasets presented in this study can be found in online repositories. The names of the repository/repositories and accession number(s) can be found below: https://www.ncbi.nlm.nih.gov/, PRJNA541796.

## Ethics Statement

The animal study was reviewed and approved by the Animal Ethics Committee of Xiamen University.

## Author Contributions

XX conceived the experiments. HL, XQ, YC, YQ, JZ, and XJ conducted the experiments. All authors assisted in the collection and interpretation of data. XX and HL have the same contribution to this paper. All authors contributed to the article and approved the submitted version.

## Funding

This research was supported by Open Research Fund Project of State Key Laboratory of Large Yellow Croaker Breeding (No.LYC2018RS04), the Natural Science Foundation of Fujian Province (# 2020J01673), the National Key Research and Development Program of China (NO. 2018YFC1406305), the Foreign Cooperation Project of Fujian Province(No.2019I1008), the Scientific Research Fund of Engineering Research Center of the Modern Industry Technology for Eel Ministry of Education(No.RE202014, RE-R202007), the Science and Technology Platform Construction of Fujian Province (No.2018N2005, 2017L3019), the NSFC (General Program No.31702384), the Scientific Research Fund of Fujian Provincial Department of Education (No.JA15292), and the open fund of the Fujian Province Key Laboratory of Special Aquatic Formula Feed (Fujian Tianma Science and Technology Group Co., Ltd.) (No.TMKJZ1907), Science and technology commissioner of fujian province (No.MinKeNong [2019] No.11, ZP2021001), the national key Research and development plan(No.2020YFD0900102).

## Conflict of Interest

XX was employed by Ningde Fufa Fisheries Company Limited and Fujian Tianma Science and Technology Group Co., Ltd. JZ was employed by Ningde Fufa Fisheries Company Limited.

The authors declare that this study received funding from Fujian Tianma Science and Technology Group Co., Ltd.. The funder had the following involvement in the study: the funder bodies were involved in the study design, interpretation of data.

The remaining authors declare that the research was conducted in the absence of any commercial or financial relationships that could be construed as a potential conflict of interest.
