# Supplementary material for: cheA, cheB, cheR, cheV, and cheY Are Involved in Regulating the Adhesion of Vibrio harveyi
Source: Front Cell Infect Microbiol. 2021 Feb 3;10:591751. doi: 10.3389/fcimb.2020.591751 (PMC7887938; doi:10.3389/fcimb.2020.591751)
Supplement: Supplementary file 1 [file DataSheet_1.docx]

Supplementary Material

# Supplementary Data

The Raw data of transcriptome for this article can be found online at https://dataview.ncbi.nlm.nih.gov/object/PRJNA541796?reviewer=pu4moklg8d0ar43hgbsmd04192

# Supplementary Figures and Tables

**Supplementary Table 1. Strains and plasmids**

| Strains or plasmids Characteristic(s) |
| --- |
| Plasmids  pACYC184 Cloning vector, Cm^R^. provided by Prof. Nie  Strains  VH6110 wild-type *V. harveyi*  control wild-type *V. harveyi* with enpty pACYC184 plasmid  *cheA*-RNAi et al Gene-silenced strain  Sm10 *thi thr leu tonA lacY supE recA* RP4-2-Tc::Mu::Km (λpir) |

**Supplementary Table 2. shRNA** **used in stable gene silencing**

| shRNA Sequences | |
| --- | --- |
| shRNA-cheA    shRNA-cheB  shRNA-cheR  shRNA-cheV  shRNA-cheY | F:5'-GATCCGCACCAAGTGATCGGTATTCTTTCAAGAGAAGAATACCGATCACTTGGTGCTTTTTTGCATG-3'  R:5'-CAAAAAAGCACCAAGTGATCGGTATTCTTCTCTTGAAAGAATACCGATCACTTGGTGCG -3'  F:5'-GATCCGCAGCAACGTGTGATTCATATTTCAAGAGAATATGAATCACACGTTGCTGCTTTTTTGCATG-3'  R:5'-CAAAAAAGCAGCAACGTGTGATTCATATTCTCTTGAAATATGAATCACACGTTGCTGCG-3'  F:5'-GATCCGCGATGACGGTATTAGAAACCTTCAAGAGAGGTTTCTAATACCGTCATCGCTTTTTTGCATG -3'  R:5'-CAAAAAAGCGATGACGGTATTAGAAACCTCTCTTGAAGGTTTCTAATACCGTCATCGCG-3'  F:5'-GATCCGCAAGCAAAGCAAATCAATCGTTCAAGAGACGATTGATTTGCTTTGCTTGCTTTTTTGCATG-3'  R:5'-CAAAAAAGCAAGCAAAGCAAATCAATCGTCTCTTGAACGATTGATTTGCTTTGCTTGCG-3'  F:5'-GATCCGCCACTCAAGCAACAAGAAAGTTCAAGAGACTTTCTTGTTGCTTGAGTGGCTTTTTTGCATG-3'  R:5'-CAAAAAAGCCACTCAAGCAACAAGAAAGTCTCTTGAACTTTCTTGTTGCTTGAGTGGCG-3' |

**Supplementary Table 3. Primers used to detect gene silencing efficiency by qRT-PCR**

| Primer Sequences | |
| --- | --- |
| VH16S  VH cheA    VH cheB  VH cheR  VH cheV  VH cheY | F: 5'-CGGTAATACGGAGGGTGCGA-3'  R: 5'-GTACTCTAGTCTGCCAGTTTCAAATGCT-3'  F: 5'-CGTTCACGCTTTGGTTTGGTTG-3'  R: 5'-CACGGTTGCTTACACCTTGGATG-3'  F: 5'-GCTCAACTGCGGCTCCAAAA-3'  R: 5'-GCGAACGCTGCGGTAAAGGT-3'  F: 5'-GGAAATAGCGGCAAACAAGCG-3'  R: 5'-GGTGAAAGTCCACGACCCAAGG-3'  F: 5'-CCAACCCACAACGCAAATCC-3'  R: 5'-GAACGCACCATAAAGGCAACG-3'  F: 5'-GAGTTGAACCAAGACCCTGCCA-3'  R: 5'-TGCTCTTTTACCGTTGCTCGC-3' |

**Supplementary Table 4. Primers used to verify transcriptome data by qRT-PCR**

| Primer Sequences | |
| --- | --- |
| VH 16S  VH ptsN    VH bioD  VH atpB  VH gppA  VH fusA  VH msrA  VH hscB  VH oppD  VH pyk  VH Hfq  VH CYC1  VH putA  VH hemA  VH psiE  VH ybaZ  VH rpsR | F: 5'-CGGTAATACGGAGGGTGCGA-3'  R: 5'-GTACTCTAGTCTGCCAGTTTCAAATGCT-3'  F: 5'- TCCAATGTGAATCGCCAGTAGAGTT -3'  R: 5'- GCTCTTGGTCTGACTGGGCATT -3'  F: 5'- GCGAAATGCCGATATTGTCTTGG -3'  R: 5'- CCCAACCTACGACTTGAAGACCG -3'  F: 5'- CTTCAAGTCGTAGGTTGGGTCGC -3'  R: 5'- ATGAAAGGTTTGATAGGTGGTGGG -3'  F: 5'- ACTCGTCAGCCGCCACAAAC -3'  R: 5'- CGCAAAGTATGGGTCGCAGC -3'  F: 5'- CTGGTGACATTGCGGCTGCT -3'  R: 5'- GGGTCTTCCGCTGCCAGTTTA -3'  F: 5'- TTTATCTTGCTGGCGGCTGC -3'  R: 5'- TTACCGACATCATTTCCTTGCTTG -3'  F: 5'- TCACAATTCCGTGAACTTCAAAAGC -3'  R: 5'- TCCATTAGGAACATTGGGTCTTGC -3'  F: 5'- CCGTTGCCTTCTCGCTGATG -3'  R: 5'- CGCTCTGGAACACCATTGACATTT -3'  F: 5'- GCGATGAACGGTGGCAAACT -3'  R: 5'- ACGTGGGAAAGAAACAGCAAGG -3'  F: 5'- ACGTCGTGAACGTATTCCAGTGTC -3'  R: 5'- CTGACGATCACCACCTTGAGGAC -3'  F: 5'- CGTGACATTGGTATCCCAGTTGAG -3'  R: 5'- AAGACGATGTTGTTTACGCCGAA -3'  F: 5'- GAATACTCGGTTGAACGCAAAGGT -3'  R: 5'- TCGTCGCTTTGGAATCGGAG -3'  F: 5'- ATTTCAACCTGTAACCGCACCG -3'  R: 5'- CCTGTCCGAGGATTTGTGGCT -3'  F: 5'- ATGCTGCTGGTTGCGATTACG -3'  R: 5'- CCGTTGACGACAAACTGCTGC -3'  F: 5'- TCATGTTGGTAAGGCGCTAGGTAA -3'  R: 5'- TTCTCAAGCTCACTTTGCCGTCT -3'  F: 5'- GCTCGTTTCTTCCGTCGTCG -3'  R: 5'- TAGGTAGCGTGAACGCTTGATAGC -3' |

## Supplementary Figures

**
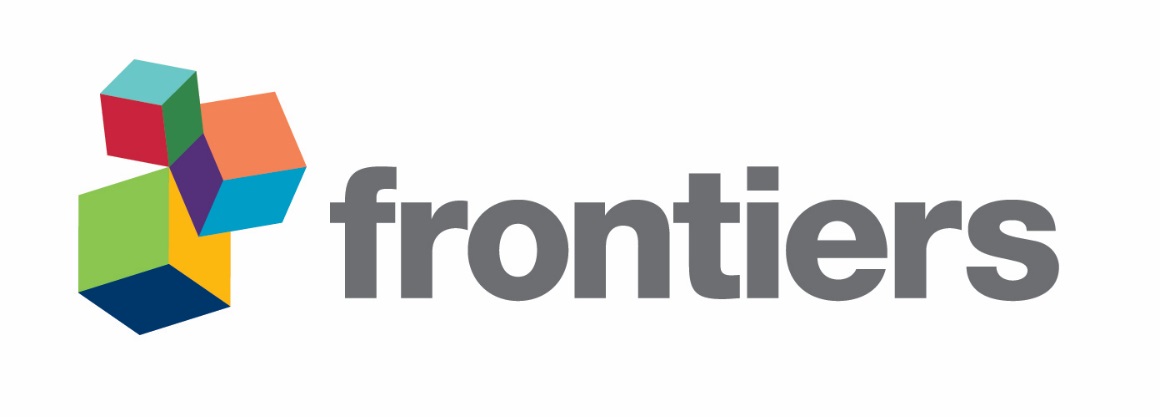
**

**Supplementary Figure 1.** Typical images of the spreading of stably silenced *Vibrio harveyi* strains and control.

**Supplementary Figure 2. [**A] The ability of the control and stressed *Vibrio harveyi* strains to form biofilms in lysogeny broth (LB) medium at 28 °C. [B] Chemotactic ability of the control and stressed *Vibrio harveyi* strains and the control to move toward the mucus. [C] Motility of the control and stressed *Vibrio harveyi* on soft agar plates. [D] Typical images of the spreading of the *Vibrio harveyi* stressed by Cu^2+^(50mg/L). [E] Typical images of the spreading of the *Vibrio harveyi* stressed by Zn^2+^(50mg/L). Data are presented as the mean ± SD (n = 3). ***P* < 0.01 compared to the control.

**Supplementary Figure 3.** GO terms for DEGs grouped into functional categories. The vertical and horizontal coordinates represent the number of genes and the GO functional terms, respectively. Red and green indicate upregulated and downregulated genes, respectively.

**Supplementary Figure 4.** RNA-seq validation of randomly selected genes analyzed by qRT-PCR. The qRT-PCR data confirmed the expression trends observed in the RNA-seq data for many genes in the *cheA*-RNAi strains.

**Supplementary Figure 5.** qRT-PCR analysis of the expression of *cheA*, *cheB*, *cheR*, *cheV* and *cheY* in wild-type *Vibrio harveyi* strains grown under different conditions of pH. Data are presented as the mean ± SD, and each treatment was performed on six independent replicates. The means of results are considered significantly different with *P* < 0.05.

**Supplementary Figure 6.** qRT-PCR analysis of the expression of *cheA*, *cheB*, *cheR*, *cheV* and *cheY* in wild-type *Vibrio harveyi* strains grown under different conditions of temperature. Data are presented as the mean ± SD, and each treatment was performed on six independent replicates. The means of results are considered significantly different with *P* < 0.05.

**Supplementary Figure 7.** qRT-PCR analysis of the expression of *cheA*, *cheB*, *cheR*, *cheV* and *cheY* in wild-type *Vibrio harveyi* strains grown under different conditions of salinity. Data are presented as the mean ± SD, and each treatment was performed on six independent replicates. The means of results are considered significantly different with *P* < 0.05.
