# Supplementary figures and images for: cheA, cheB, cheR, cheV, and cheY Are Involved in Regulating the Adhesion of Vibrio harveyi
Source: Front Cell Infect Microbiol. 2021 Feb 3;10:591751. doi: 10.3389/fcimb.2020.591751 (PMC7887938; doi:10.3389/fcimb.2020.591751)

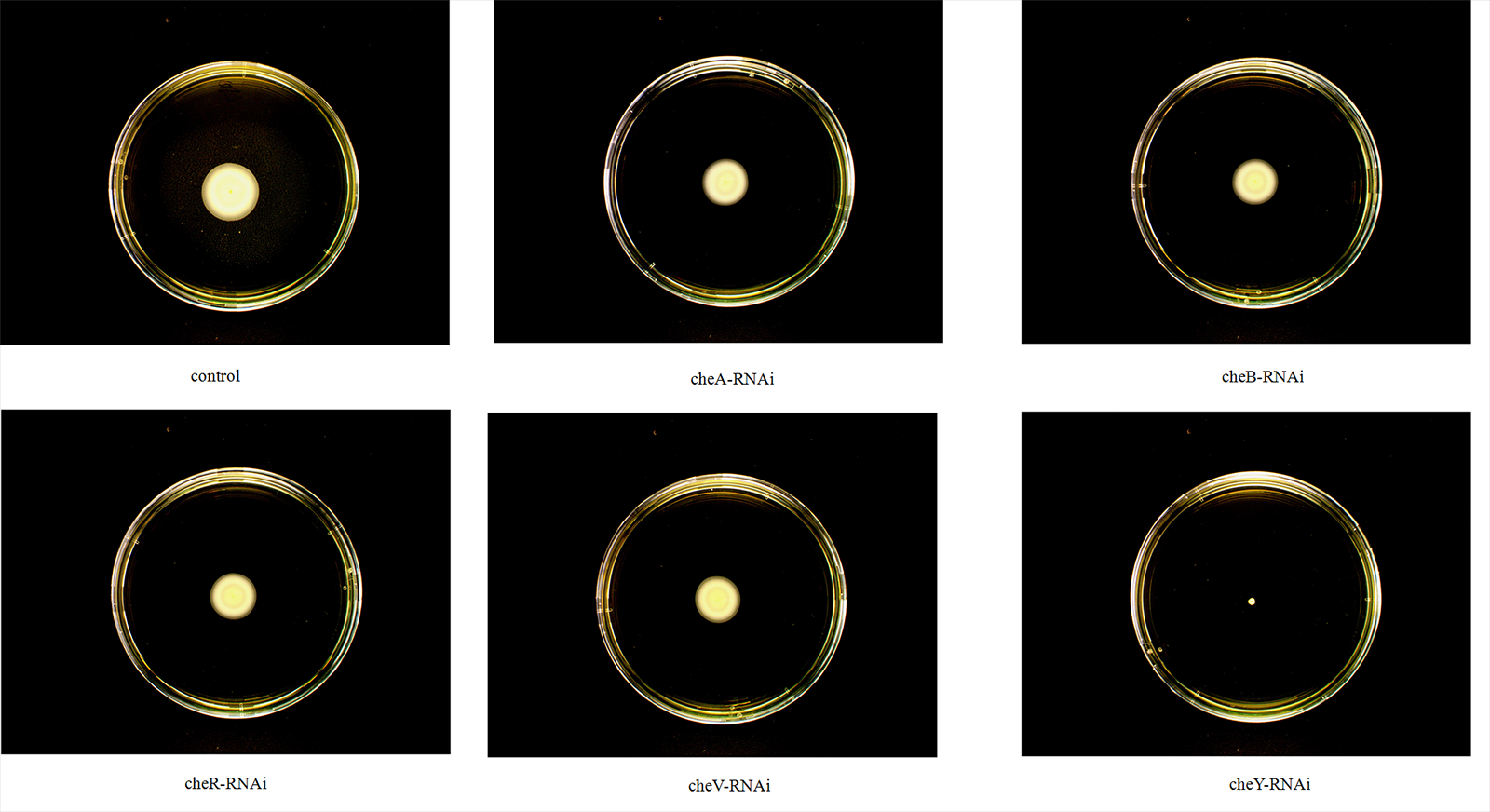

Supplement: Supplementary file 2 [file Image_1.tif]

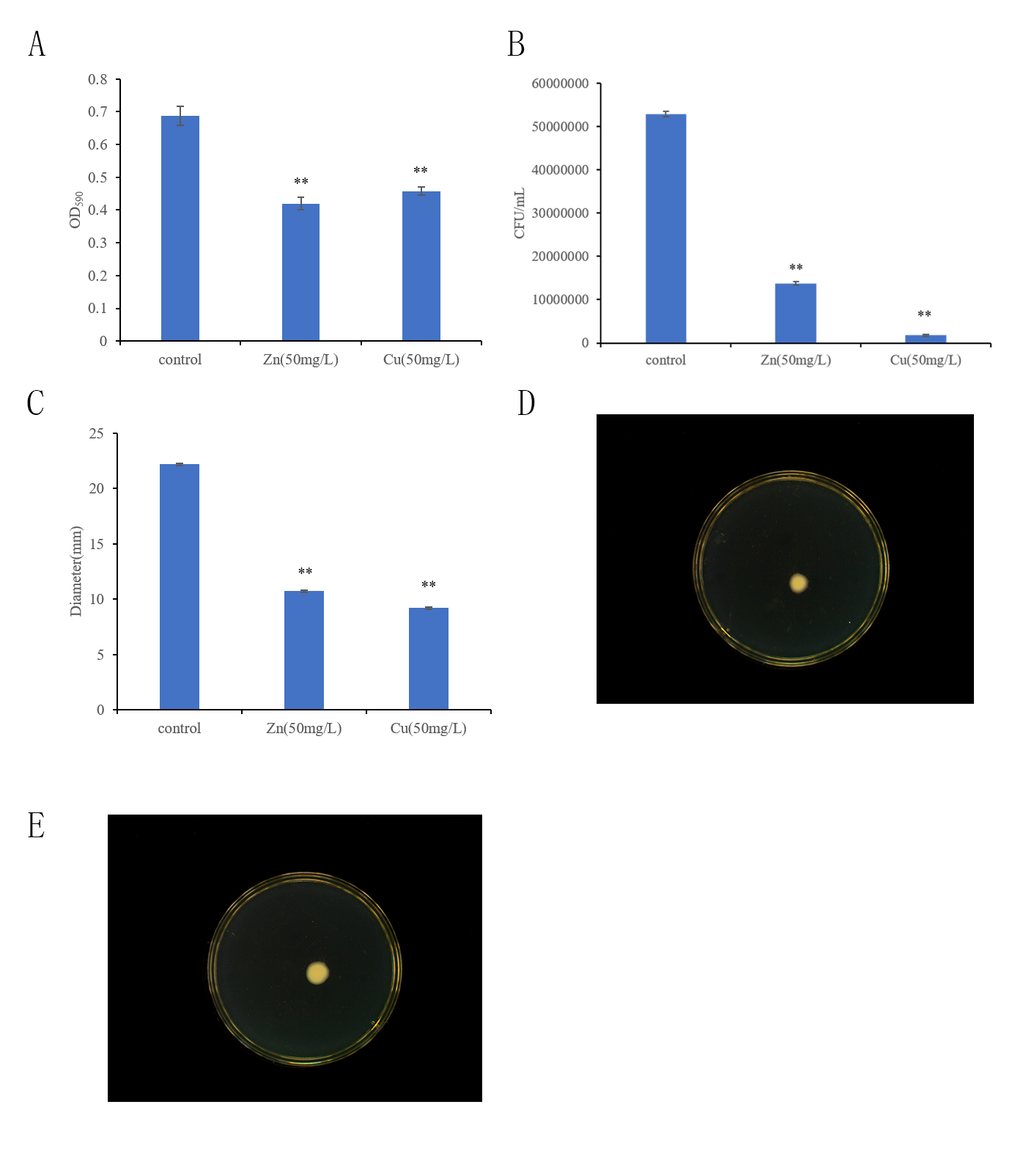

Supplement: Supplementary file 3 [file Image_2.tif]

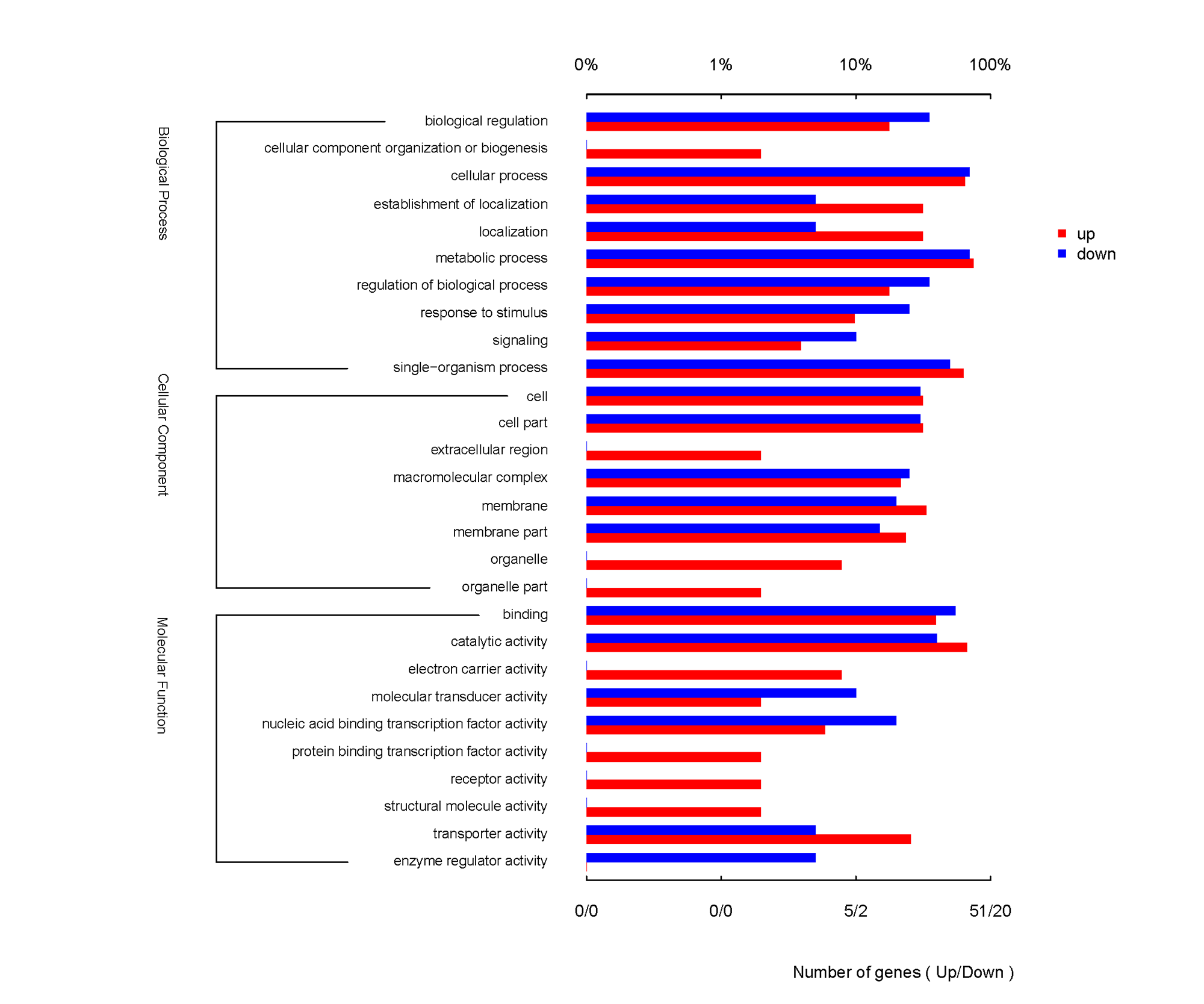

Supplement: Supplementary file 4 [file Image_3.tiff]

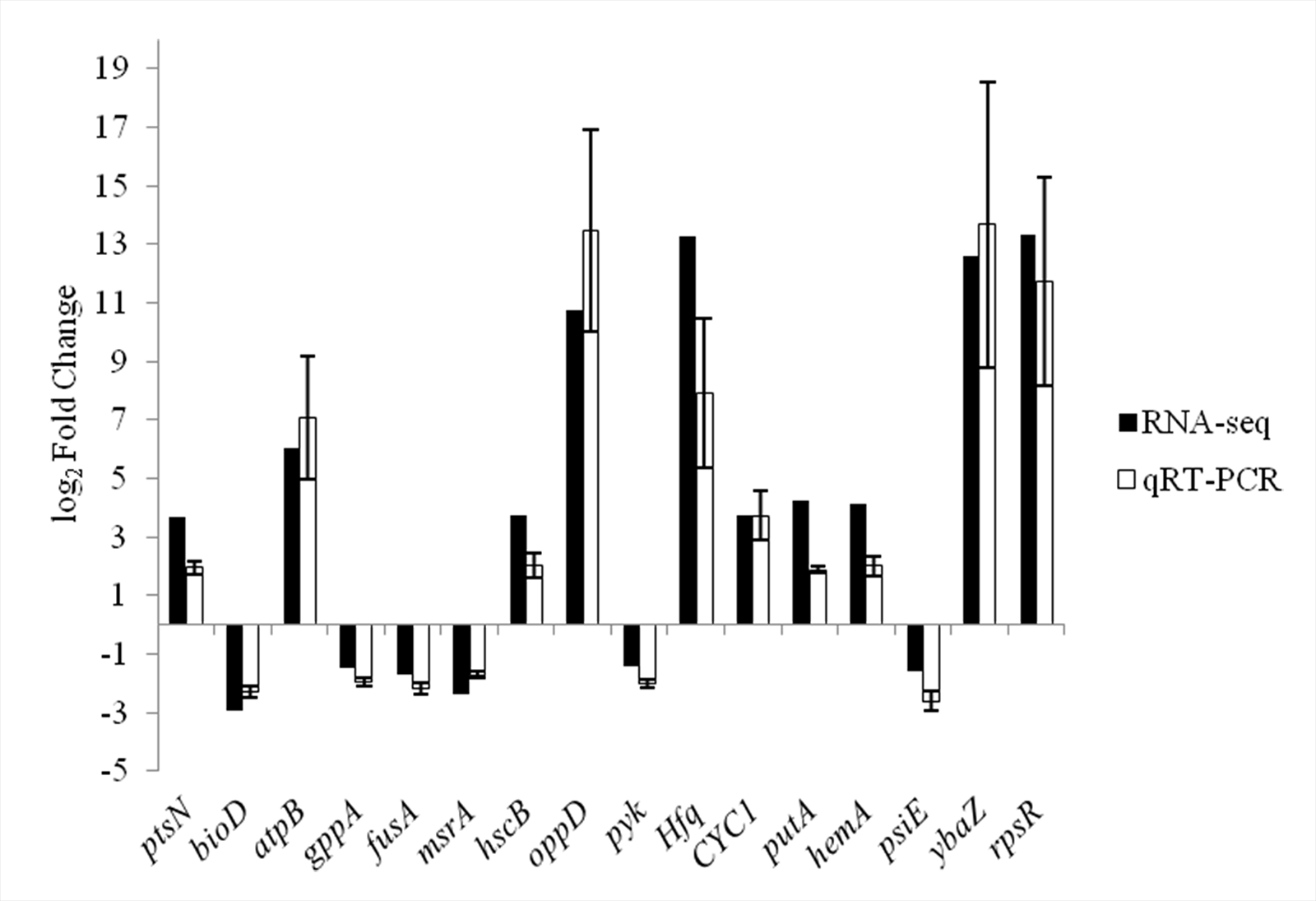

Supplement: Supplementary file 5 [file Image_4.tif]

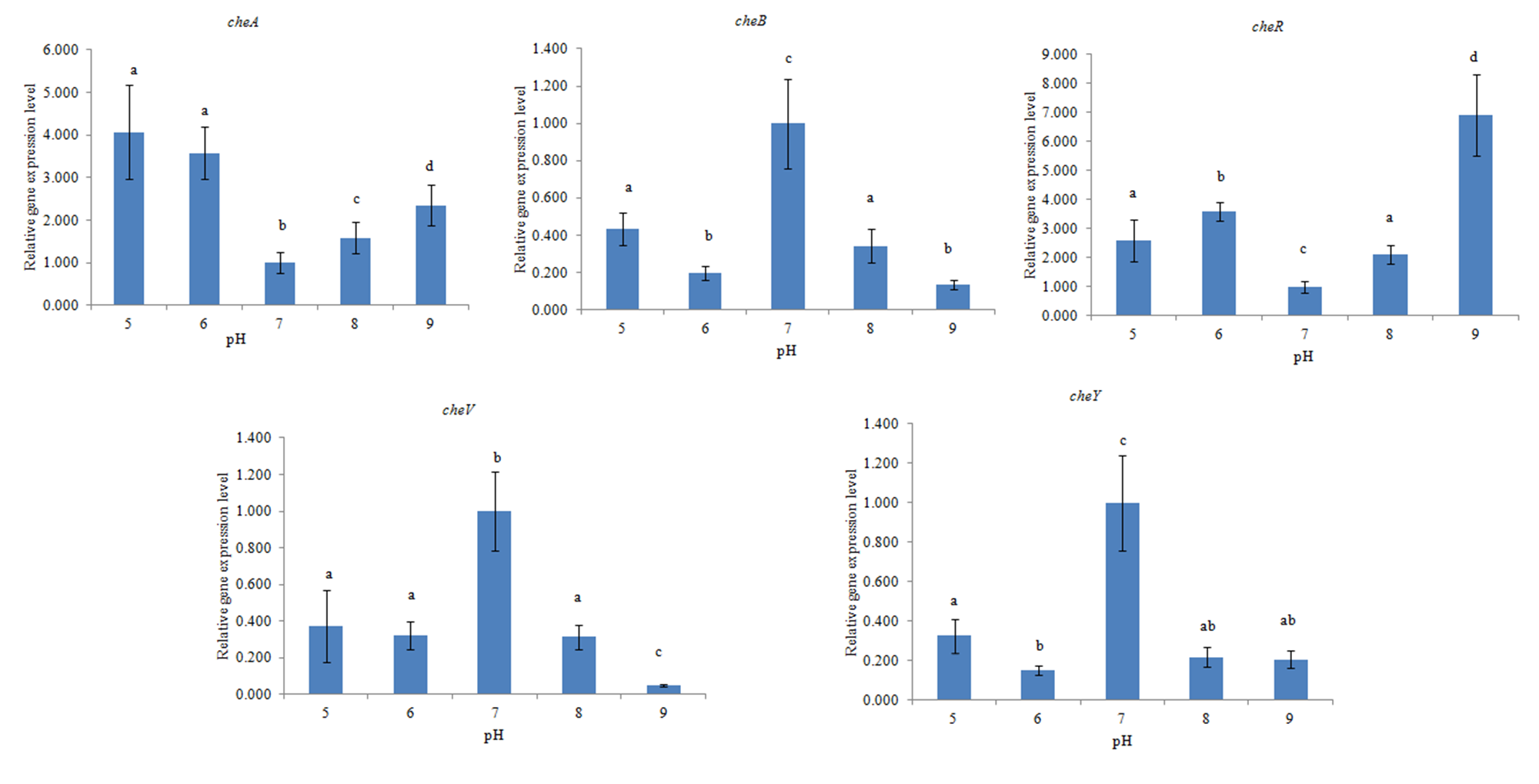

Supplement: Supplementary file 6 [file Image_5.tif]

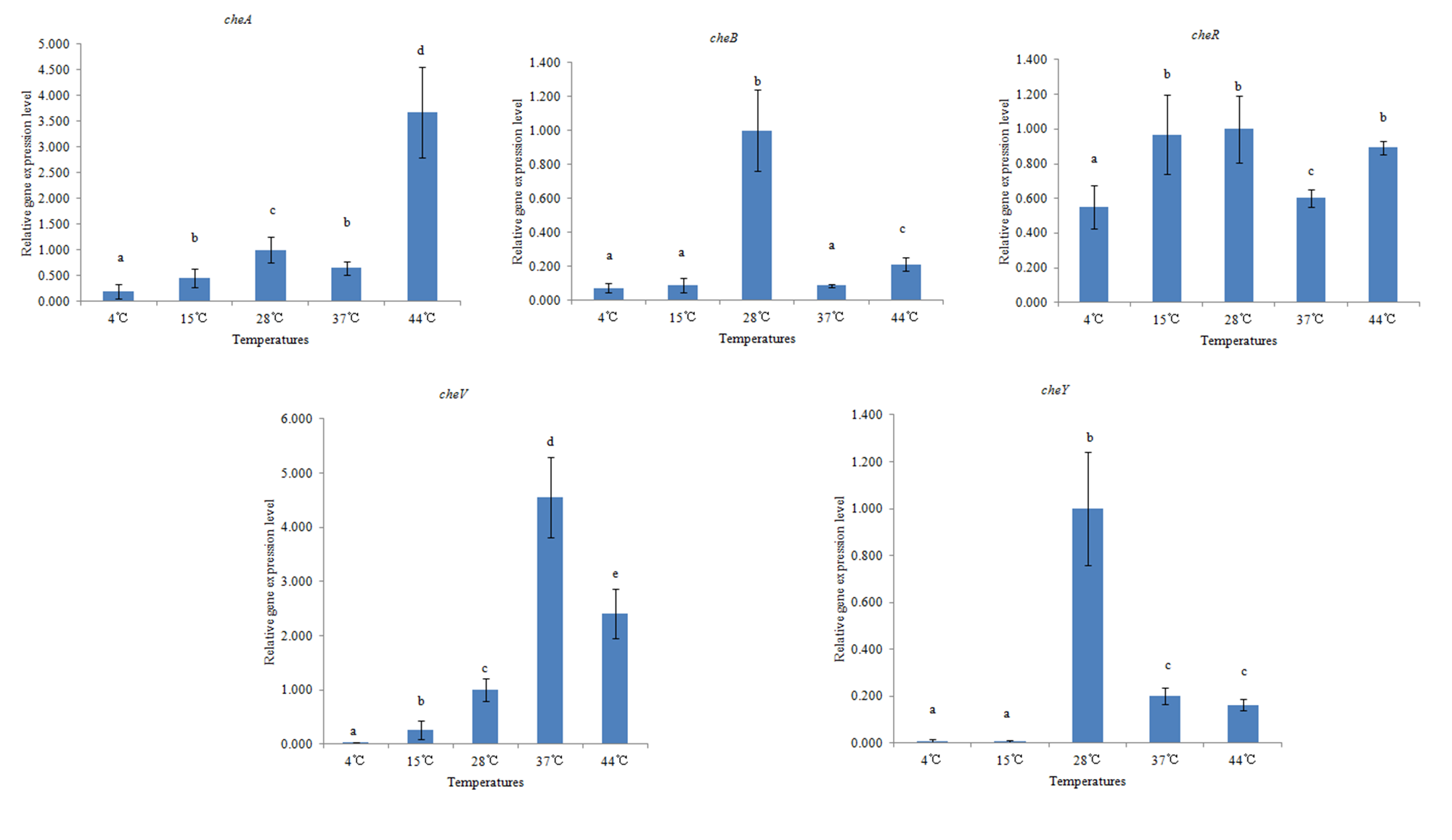

Supplement: Supplementary file 7 [file Image_6.tif]

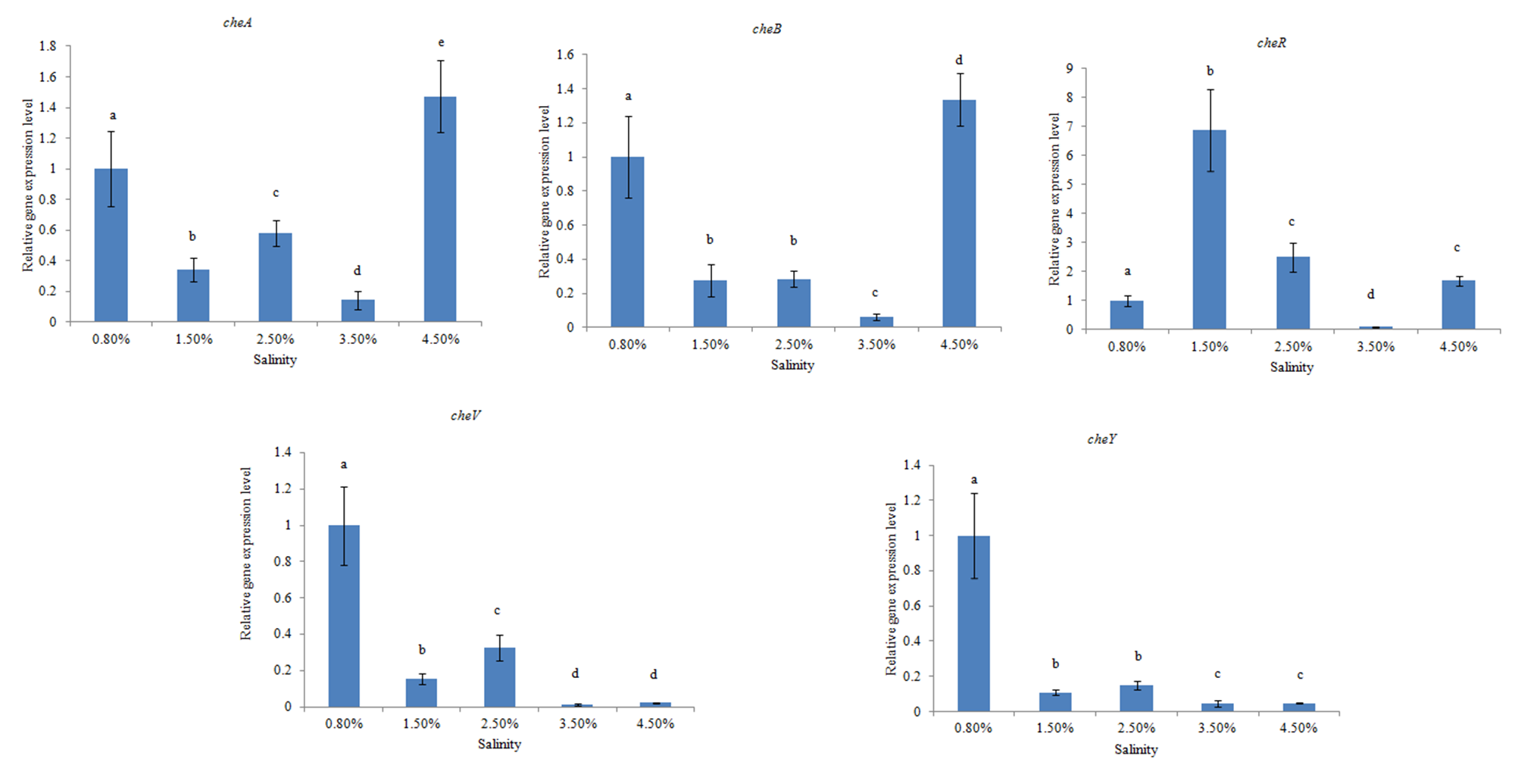

Supplement: Supplementary file 8 [file Image_7.tif]
